# The impact of adenomyosis localization in myometrium on fertility and pregnancy outcomes: a narrative systematic review of the literature

**DOI:** 10.3389/frph.2026.1748474

**Published:** 2026-03-16

**Authors:** Georgios Kolovos, Theodoros Kalampokas, Ioannis Dedes, Nikolaos Vlahos, Michael Mueller

**Affiliations:** 1 Department of Obstetrics and Gynecology, Inselspital, University Hospital of Bern, Bern, Switzerland; 2 Department of Obstetrics and Gynecology, Bürgerspital Solothurn, Solothurn, Switzerland; 3 Second Department of Obstetrics and Gynecology, Aretaieion University Hospital, National and Kapodistrian University of Athens, Athens, Greece; 4 Department of Obstetrics and Gynecology, Kantonsspital Glarus, Glarus, Switzerland

**Keywords:** adenomyosis, extrinsic adenomyosis, infertility, intrinsic adenomyosis, medically assisted reproduction, pregnancy

## Abstract

**Background:**

Adenomyosis can be categorized using various systems based on its extent, location, and severity. Depending on its location within the endometrium adenomyosis can be classified as intrinsic (inner myometrium) or extrinsic (outer myometrium). This narrative systematic review aims to comprehensively analyze existing literature to determine whether a significant correlation exists between the localization of adenomyosis within the uterus and infertility. Additionally, it evaluates the impact of intrinsic and extrinsic adenomyosis on outcomes of medically assisted reproduction, with a particular focus on clinical pregnancy and live birth rates.

**Methods:**

We conducted a narrative systematic literature search in PubMed, Scopus, ScienceDirect, Cochrane and Embase Databases from inception to June 2024. The search was based on the key words Keywords such "infertility", "assisted reproductive technologies (ART)", "adenomyosis," and "adenomyosis uteri". All English full-text prospective and retrospective observational and interventional studies with at least ten patients that described reproductive outcomes of women diagnosed with intrinsic or extrinsic adenomyosis; either through sonographic or through MRI modalities, were included. This protocol has been registered with PROSPERO (CRD42023479565).

**Results:**

A total of 5608 articles were initially identified from the search strategy with only 9 of them included in the review. Among them our review incorporated data from six retrospective studies and three prospective observational studies. Our findings revealed noteworthy difference among the different patient groups. Extrinsic adenomyosis was consistently associated with a higher prevalence of endometriosis, particularly deep infiltrating endometriosis (DIE) and ovarian endometriomas, with reported association rates reaching up to 89%. Additionally, extrinsic adenomyosis correlated with a higher prevalence of primary infertility (41.3%), compared to intrinsic adenomyosis (20.7%). In contrast, intrinsic adenomyosis exhibited a stronger association with recurrent pregnancy loss (RPL) and secondary infertility. Regarding assisted reproductive technology (ART) outcomes, available data remain limited and safe conclusions cannot be made.

**Conclusions:**

Despite the limited number of available studies and the heterogeneity in definitions of mixed, diffuse, and advanced adenomyosis, the existing evidence suggests that adenomyosis subtypes differ in clinical presentation according to their localization within the myometrium and may exert distinct effects on fertility, pregnancy, and ART outcomes. With the continuous development of our diagnostic tools and minimal invasive therapeutic methods this insight could empower clinicians to provide accurate information to their patients with adenomyosis according to the classification and potentially modify therapeutic plans based on clinical suspicions.

**Systematic Review Registration:**

PROSPERO CRD42023479565.

## 1 Introduction

Adenomyosis, a complex and enigmatic gynecological condition, is garnering growing interest in women's health research. It is characterized by the infiltration of endometrial glands and stroma into the uterine muscular tissue, presenting a unique challenge in clinical practice. Sampson was the first who tried to classify the adenomyosis with an etiological theory and described the disease as “adenomyoma” (1). The most prominent pathophysiologic theories regarding the formation of adenomyosis are the following; 1) the microtrauma of endometrial-myometrial interface and the tissue injury and repair mechanism (TIAR), 2) the theory of invasion of endometrial basalis into the myometrium, 3) the *de novo* metaplasia theory or Mullerian remnant theory and last but not least 4) the outside to inside theory (2). The prevalence of adenomyosis is substantial, particularly evident in examinations of hysterectomy specimens. In cases where dysmenorrhea and abnormal uterine bleeding are the predominant symptoms, prevalence rates escalate significantly, ranging from 30% to 59% (3, 4). Recent advancements in imaging technologies, particularly transvaginal ultrasound and magnetic resonance imaging, have ushered in a transformative era in adenomyosis diagnosis enabling earlier and more accurate disease identification, fundamentally altering the diagnostic landscape. Younger women of reproductive age are now being diagnosed, often presenting with a diverse array of symptoms. In women undergoing assisted reproductive technologies (ART), adenomyosis is present in approximately 20% to 25% of cases (5, 6).

**Table 1:** Demographic data, classification on adenomyosis lesions.

Study	General characteristics				Imaging			
	Country study type	Duration	Age (years)	BMI (kg/m^2^)	MRI (0: no, 1: yes)	US (0: no, 1: yes)	Type (Intrinsic, Extrinsic/FOAM, Diffuse)	Localization (anterior, posterior, fundus)
Iwasawa et al., (18)	Japan rertrospective	January 2012 to December 2016	37 (30–39)	19.9 (18.1–23.3)	1	0	9 extrinsic	1 circumferential, 1 anterior wall and 7 on the posterior wall
			35 (31–36)	23.5 (19.5–25.8)	1	0	3 intrinsic	1 anterior wall and 2 on the posterior wall
			36 (26–41)	23 (18.1–32.5)	1	0	40 advanced cases	8 circumferential, 4 anterior wall and 28 on the posterior wall
Marques ALS et al., (16)	Brasil prospective	January 2018 to December 2018	29.4 ± 5.7	n/a	0	1	11 extrinsic	n/a
			30.5 ± 5.6	n/a	0	1	22 intrinsic	n/a
			36.7 ± 2.6	n/a	0	1	11 diffuse	n/a
Kobayashi H et al., (15)	Japan prospective	April 2008 to March 2018	43 (21–52)	21.2 (16.0–36.0)	1	0	78 extrinsic	n/a
			44 (33–55)	22.5 (13.0–39.0)	1	0	74 intrinsic	n/a
Bourdon M et al., (13)	France rertrospective	May 2005 to May 2018	n/a	n/a	1	0	109 extrinsic	n/a
			n/a	n/a	1	0	78 diffuse	n/a
			n/a	n/a	1	0	61 FAOM & diffuse	n/a
Bourdon M et al., (17)	France retrospective	June 2015 to July 2018	n/a	n/a	1	0	intrinsic 64	n/a
			n/a	n/a	1	0	123 FOAM (extrinsic)	anterior FOAM 20/123, posterior FOAM 114/123
Exacoustos C et al., (19)	Italy retrospective	January 2018 to Ferbruary 2023	n/a	n/a	0	1	15 intrinsic of whom 9 focal and 6 diffuse	n/a
			n/a	n/a	0	1	42 extrinsic of whom 13 focal and 29 diffuse	n/a
			n/a	n/a	0	1	23 both intrinsic and extrinsic	n/a
Valdés-Bango M et al., (14)	Spain prospective	January 2021 to December 2022	41.2 ± 6.1	24.7 ± 4.5	0	1	353 intrinsic	35 (16.5%) anterior while 77 (83.5%) posterior
			39.7 ± 6.2	23.5 ± 4.5	0	1	152 extrinsic	35 (23.0%) anterior while 117 (77.0%)posterior
Han X et al., (11)	China rertrospective	January 2015 to October 2020	42.10 ± 6.19	n/a	1	0	184 intrinsic	n/a
			36.93 ± 6.29	n/a	1	0	208 extrinsic	n/a
Bourdon M et al., (10)	France retrospective	May 2005 to May 2018	33.8 ± 5.2	23.17 ± 4.14	1	0	78 intrinsic	n/a
			31.9 ± 4.6	21.8 ± 3.13	1	0	109 extrinsic	n/a

**Table 2:** Endometriosis characteristics, #ENZIAN classification, prior endometriosis surgery.

Publication	Type	Endometriosis	P	O	T	A	B	C	FBladder	Prior endometriosis surgery
Iwasawa et al., (18)	9 extrinsic	4 (44%)	n/a	9 (100%)	n/a	n/a	n/a	n/a	n/a	7/9 endometrioma surgery
	3 intrinsic	0	0	0	0	0	0	0	0	1/3 endometrioma surgery
	40 advanced cases	24 (60%)	n/a	28 (70%)	n/a	n/a	n/a	n/a	n/a	13/40 endometrioma surgery
Kobayashi H et al., (15)	78 extrinsic	DIE and/ or SUP 50 (64.1%) *p* < 0.05	n/a	50 (64.1%) *p* < 0.05	n/a	n/a	n/a	n/a	n/a	n/a
	74 intrinsic	DIE and/ or SUP 5 (6.8%)	n/a	4 (5.4%)	n/a	n/a	n/a	n/a	n/a	n/a
Valdés-Bango M et al., (14)	353 intrinsic	DIE 264 (74.8%)	n/a	157 (44.5%)	n/a	45 (12.7)	243 (68.8%)	144 (40.8%)	10 (2.8%)	180 (51.1%) of whom 66 (18.7%) DIE surgery
	152 extrinsic	DIE 132 (86.8%) *p* = 0.003	n/a	75 (49.3%), *p* > 0.05	n/a	13 (8.6), *p* > 0.05	119 (78.3%), *p* < 0.05	65 (42.8%) *p* > 0.05	13 (8.6%), *p* < 0.05	79 (52.0%) of whom 26 (17.1%) DIE surgery
Han X et al., (11)	184 intrinsic	DIE 13 (7.1%)	n/a	29 (15.8%), *p* < 0.05	n/a	n/a	n/a	n/a	n/a	n/a
	208 extrinsic	DIE 83 (39.9%), *p* < 0.05	n/a	145 (69.7%)	n/a	n/a	n/a	n/a	n/a	n/a
Bourdon M et al., (10)	78 intrinsic	48 (62%) of whom 24 (31%) had DIE	n/a	15 (20%)	n/a	n/a	n/a	n/a	n/a	19 (24%)
	109 extrinsic	105 (96.3%) *p* < 0.05 of whom 97 (89.0%) had DIE *p* < 0.05	n/a	4 (3.7%)	n/a	n/a	n/a	n/a	n/a	52 (47.7%) *p* = 0.001

n/a, not available; DIE, deep infiltrating endometriosis; SUP, superficial peritoneal endometriosis.

**Table 3:** Data regarding infertility, ART application.

General characteristics		Infertility						
Publication	Type	Infertility and cause (when available)	Gravidity	Parity	Curettage/hysteroscopy	Pregnancy loss (miscarriage)	Prior ART	Comments
Iwasawa et al., (18)	9 extrinsic	Tubal factor: 1 patient, anovulation: 0, mixed: 3, unexplainded: 1, endometriosis 4	4/9 (44.4%)	3/9	n/a	1/9 (11.1%)	27 Embyotransfers	
	3 intrinsic	Tubal factor: 0, anovulation: 1, mixed: 1, unexplainded: 1, endometriosis 0	2/3 (66.7%)	0	n/a	2/3 (66.7%)	9 Embyotransfers	
	40 advanced cases	Tubal factor: 2 patients, anovulation: 0, mixed: 9, unexplainded: 5, endometriosis 24	17/ 40 (42.5%)	7/40	n/a	13/40 (32.5%)	100 Embyotransfers	
Marques ALS et al., (16)	11 extrinsic	9 (81.8%)	n/a	n/a	n/a	n/a	n/a	In total 3 patients had at least one pregnancy (7%)
	22 intrinsic	16 (72.7%)	n/a	n/a	n/a	n/a	n/a	
	11 diffuse	10 (100%)	n/a	n/a	n/a	n/a	n/a	
Kobayashi H et al., (15)	78 extrinsic	25/78 (32.1%)	Gr.0: 27 (34.6%) Gr.1: 16 (20.5%), Gr > 1:35 (44.9%)	P.0: 31 (41%) P.1: 21 (26.9%), *P* > 1:25 (32.1%)	0: 68 (87.2%), 1: 7 (9%), >1: 3 (3.8%)	n/a	n/a	
	74 intrinsic	3/74 (4.1%) ***p*** **>** **0.05**	Gr.0: 19 (25.7%) Gr.1: 10 (13.5%), Gr > 1:45 (60.8%)	P.0: 18 (24.3%) P.1: 13 (17.6%), *P* > 1:43 (58.1%)	0: 39 (52.7%), 1: 19 (25.7%), >1: 16 (21.6%)	n/a	n/a	
Bourdon M et al., (13)	109 extrinsic	prim. Infertility 28 (25.6%) sec.Infertility 9 (8.3%)	n/a	n/a	n/a	n/a	n/a	
	78 diffuse	prim. Infertility 7 (9%) sec.Infertility 9 (11.5%)	n/a	n/a	n/a	n/a	n/a	
	61 FAOM & diffuse	prim. Infertility 14 (22.9%) sec.Infertility 8 (13.1.%)	n/a	n/a	n/a	n/a	n/a	
Bourdon M et al., (10)	78 intrinsic	total 16 (20.5%), prim. infertility 7 (9%), sec.infertility 9 (12%)	26 (33%)	*P* 1: 17 (22%), *P*=>2: 27 (35%)	n/a	12 (23%) ***p*** **>** **0.05**	n/a	
	109 extrinsic	total 38 (34%) ***p*** **<** **0.05,** prim.infertility 28 (25.1%),**<0.05 s** infertility 9 (8.3%)	81 (74.3%) ***p*** **<** **0.05**	*P* 1: 5 (4.6%), *P*=>2: 9 (8.3%) ***p*** **<** **0.05**	n/a	9 (32.1%)	n/a	
Exacoustos C et al., (19)	15 intrinsic of whom 9 focal and 6 diffuse	n/a	n/a	n/a	n/a	11 out of 15 of whom 9 with focal and 2 with diffuse	n/a	
	42 extrinsic of whom 13 focal and 29 diffuse	n/a	n/a	n/a	n/a	16 out of 42 of whom 3 focal and 13 diffuse	n/a	
	23 both intrinsic and extrinsic	n/a	n/a	n/a	n/a	13 out of 23	n/a	
Valdés-Bango M et al., (14)	353 intrinsic	160 (45.3%)	n/a	nullipartity in 173 patients (49.0%)	n/a	n/a	n/a	
	152 extrinsic	74 (48.7%) ***p*** **>** **0.05**	n/a	nulliparity in 93 patients (61.2%) ***p*** **<** **0.05**	n/a	n/a	n/a	
Han X et al., (11)	184 intrinsic	total 38 (20.7%) ***p*** **<** **0.05**, prim.infertility 9/38 (23.7%) sec. infertility 29/38 (76.3%)	n/a	n/a	n/a	n/a	31.6% (12/38)	
	208 extrinsic	total 86 (41.3%**),** prim. infertility 50/86, 58.1%) sec. infertility 36/86 (41.9%)	n/a	n/a	n/a	n/a	31.4% (27/86)	

n/a, not available. *p*-value < 0.05 was considered statistically significant.

**Table 4:** ART outcomes.

General characteristics		IVF/ ICSI character istics											IVF outcomes			
Publication	Type	Prior ART	Oocyte retrieval cycles	Number of oocytes retrieved	Type of ovarian stimulation	IVF	ICSI	Mixed	Number of ET cycles	ET type	Embryo quality	Clinical pregnancy rate/ET, *n* (%)	Pregnancy loss/clinical pregnancy, *n* (%)	Pregnancy loss after 12 weeks, *n* (%)	Live birth rate/ ET, *n* (%)	Comments
Iwasawa et al., (18)	9 extrinsic	27 ET	2 (1−4)	4 (1−11)	short 4/21, long 11/21, Ultralong 2/21, GnRH antagonist 3/21, other 1/21	14/21	6/21	1/21	27	Fresh 11/27, frozen-thawed 16/27	Good 16/27	9/27 (33.3%)	3/9 (33.3%)	0/3	6/27 (22.2%)	
	3 intrinsic	9 ET	1	8 (8–14)	short 0/3, long 1/3, Ultralong 1/3, GnRH antagonist 1/3, other 0/3	1/3	2/3	0/3	9	Fresh 1/9, frozen-thawed 8/9	Good 9/9	2/9 (22.2%)	1/2 (50%)	0/1	1/9 (11.1%)	
	40 advanced cases	100 ET	2 (1–9)	2 (0–15)	Short 49 (111), long 26/111, Ultralong 16/111, GnRH antagonist 15/ 111,	67/106	35/ 106	4/106	100	Fresh 49/100, frozen-thawed 51/100	Good 77/100	25/100 (25%)	16/25 (64%)	4/16 (25%)	9/100 (9%)	
Bourdon M et al., (17)	Intrinsic 64	1	n/a	n/a	a GnRH antagonist protocol; a long agonist protocol; and a short agonist protocol	n/a	n/a	n/a	n/a	n/a	n/a	n/a	n/a	n/a	n/a	number of oocyte retrieval cycles in total: total number 346, Cycle 1: 202/346 (58.4), Cycle 2:95/346 (27.4), Cycle 3: 38/346 (11.0), Cycle 4: 11/346 (3.2) in total, mean number IVF/ICSI cycles per woman was 1.7 +/- 0.8 in total,
	123 FOAM (extrinsic)	1	n/a	n/a	a GnRH antagonist protocol; a long agonist protocol; and a short agonist protocol	n/a	n/a	n/a	n/a	n/a	n/a	n/a	n/a	n/a	n/a	Cumulative early pregnancy loss rate per woman 16/140 (11.4) in total, no pregnancy: 35/86 (total of patients who did not become pregnant) (40.8%), pregnancy 29/ 116 (25%)
Han X et al., (11)	184 intrinsic	31.6% (12/38) had ART	n/a	n/a	n/a	n/a	n/a	n/a	n/a	n/a	n/a	25% (3/12) was the success rate	n/a	n/a	n/a	
	208 extrinsic	31.4% (27/86) had ART	n/a	n/a	n/a	n/a	n/a	n/a	n/a	n/a	n/a	51.9% (14/27) was the success rate	n/a	n/a	n/a	

**Table 5:** Perinatal and pregnancy outcomes.

General characteristics		Obstetrical complications						
Publication	Type (Intrinsic, Extrinsic/FOAM, Diffuse)	Caesarean section	Number of induced abortions	Preterm labor	Cervical incompetency	Placenta previa	Fetal growth restriction	Preeclampsia
Iwasawa et al., (18)	9 extrinsic	3/6 (50%)	n/a	2/6 (33.3%)	1/6 (16.7%)	0/6	0/6	0/6
	3 intrinsic	0/1	n/a	0/1	0/1	0/1	0/1	0/1
	40 advanced cases	7/9 (77.8%)	n/a	1/9 (11.1%)	1/9 (11.1%)	1/9 (11.1%)	1/9 (11.1%)	1/9 (11.1%)
Kobayashi H et al., (15)	78 extrinsic	0:65 (83.3%), 1: 9 (11.5%), >1: 4 (51.3%)	0: 68 (87.2), 1: 7 (9.0), >1: 3 (3.8)	n/a	n/a	n/a	n/a	n/a
	74 intrinsic	0:67 (90.5%), 1:4 (5.4%), >1: 3 (4.1%)	0: 39 (52.7), 1: 19 (25.7), >1: 16 (21.6)	n/a	n/a	n/a	n/a	n/a

**Table 6:** Types of adenomyosis (intrinsic and extrinsic) and the corresponding outcomes.

	Corresponding outcomes	Intrinsic adenomyosis (Inner myometrium)	Extrinsic adenomyosis (outer myometrium; often FOAM)
Endometriosis	Association with endometriosis (overall)	Low prevalence	High prevalence
	Ovarian endometriomas	Rare or absent	Very frequent
	Deep infiltrating endometriosis (DIE)	Rare	Common
	Previous endometriosis surgery	Less frequent	More frequent
Infertility	Primary infertility	Less common	More common; independently associated (FOAM)
	Secondary infertility	More frequent than primary	Less frequent than primary
RPL	Recurrent pregnancy loss (RPL)	More frequently observed	Less frequent
ART	ART utilization (IVF/ICSI)	Similar rates to extrinsic	Similar rates to intrinsic
	ART success/live birth rates	Lower or comparable; limited data	Trend toward higher success in some studies, but inconsistent and not statistically significant
	Pregnancy loss after ART	Variable; limited data	Variable; limited data
perinatal outcomes	Perinatal outcomes	No clear differences demonstrated	No clear differences demonstrated
	Cesarean section rate	Similar to extrinsic	Similar to intrinsic

The initial attempts to describe adenomyosis using ultrasonography can be traced back to the late 1970s and the 1980s (7, 8). Adenomyosis tends to be classified by different systems, considering parameters such as extent, location and severity. In 2010 an international collaboration, the International Endometrial Tumor Analysis (IETA) group, was established to define the sonographic characteristics of various endometrial and intrauterine lesions, including adenomyosis, and to standardize the terminology used in diagnostic imaging (9). Additionally, classifications can be based on histopathological and imaging characteristics. The Kishi Classification served as a pioneering initial effort that inspired physicians to delve deeper into the localization of adenomyosis, distinguishing between two distinct types: adenomyosis of the inner myometrium, which does not impact the outer structures of the uterus such as the serosa and the adenomyosis of the outer myometrium, which does no impact the inner structures such as the endometrium. Many publications have since insisted on distinguishing between these two different types of adenomyosis, which can be easily diagnosed by ultrasound or MRI.

Traditionally the International Federation of Gynecology and Obstetrics (FIGO) has recognized adenomyosis as a distinct entity within the PALM-COEIN classification system for abnormal uterine bleeding (AUB). Recent findings suggest that the various subtypes of adenomyosis—particularly intrinsic vs. extrinsic and diffuse vs. focal—possess distinct origins and clinical characteristics. Intrinsic adenomyosis, which is more prevalent in older patients and frequently linked to abnormal uterine bleeding (AUB) and previous uterine surgeries, differs from extrinsic adenomyosis. The latter is generally observed in younger, nulligravid women and is often associated with deep infiltrating endometriosis and primary infertility (10). In a retrospective study of 468 patients diagnosed with adenomyosis through MRI examination it could be shown that proportion of dysmenorrhea and menorrhagia was higher in the intrinsic than in the extrinsic subtype (11). Patients presenting with infertility are increasingly being diagnosed with adenomyosis, suggesting a potential link between this condition and subfertility or infertility. The study by Mishra et al. was the first systematic review to assess the prevalence of isolated adenomyosis and adenomyosis coexisting with endometriosis and/or fibroids in women with subfertility. The pooled prevalence rates were 10% for isolated adenomyosis, 1% for adenomyosis with coexisting fibroids, 6% for adenomyosis with coexisting endometriosis, and 7% for adenomyosis with both endometriosis and fibroids (12). It remains unclear which type of adenomyosis has the most detrimental impact on fertility outcomes. Therefore, it is crucial to examine the different phenotypes of adenomyosis to draw more accurate conclusions. In this narrative systematic review, we explore the evidence regarding the impact of intrinsic and extrinsic adenomyosis on infertility, assisted reproduction outcomes and review the adverse obstetric outcomes associated with these subtypes of the disease in the context of personalized and precision medicine.

## 2 Methods

### 2.1 Study protocol, search strategy

**Figure 1:**
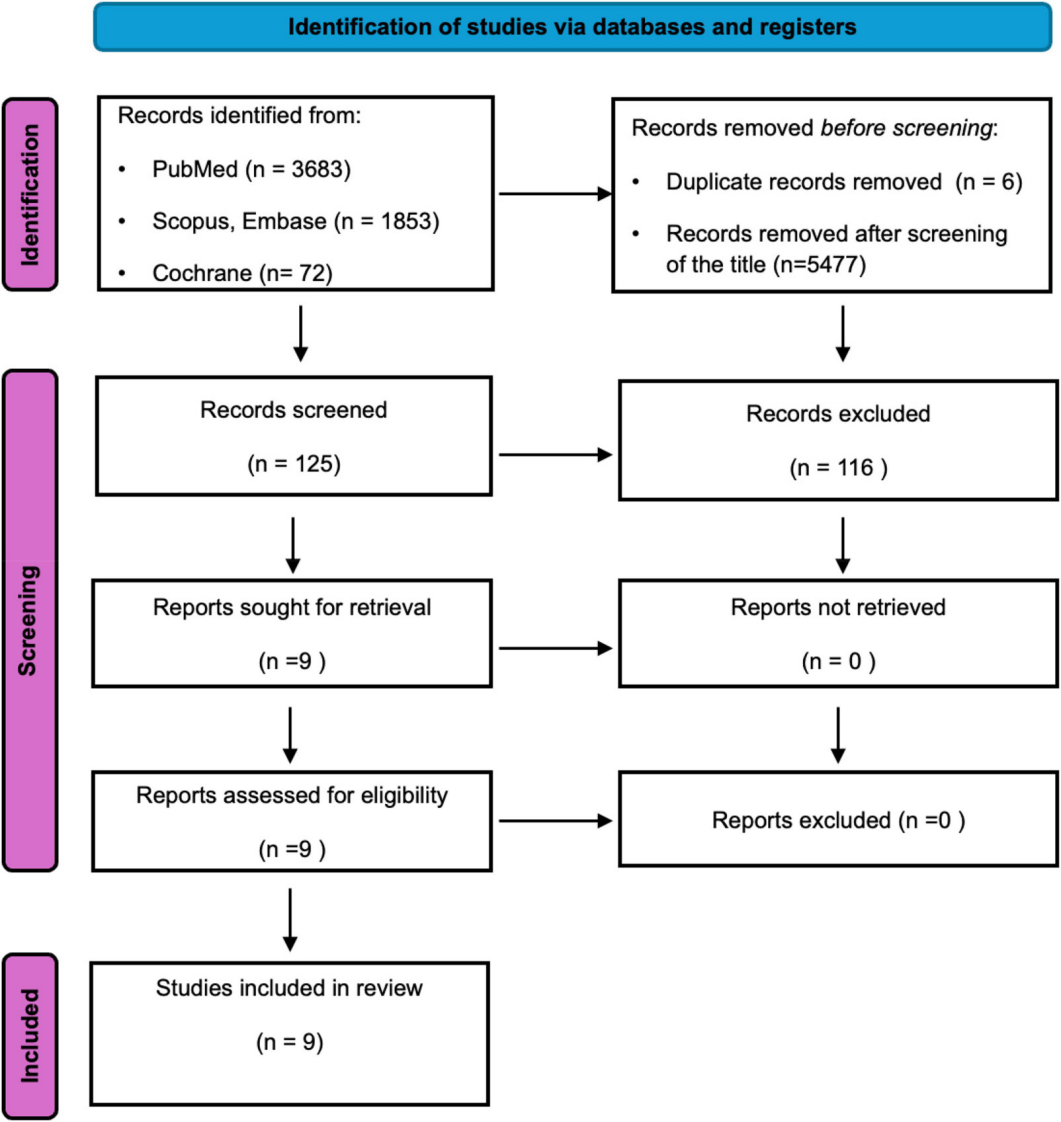
Flowchart for study selection according to the PRISMA guidelines.

The review was performed in accordance with the guidelines of the preferred Reporting Items for Systematic Reviews and Meta-Analysis (PRISMA, Figure 1). The study protocol was registered and accepted by the International Prospective Register of Systematic Reviews (CRD42023479565). Reports of women with adenomyosis and extensive description of their disease according to Kishi classification were included to the study. We conducted a comprehensive literature search to identify eligible studies using electronic databases, specifically PubMed, Scopus, Embase and ScienceDirect and Cochrane. PubMed search was conducted using the algorithm (((fertility) OR (infertility) OR (sub fertility) OR (assisted reproductive technology) OR (ART) OR (ivf) OR (*in vitro* fertilization)) AND (adenomyosis)), while for the rest databases keywords such as “infertility”, “assisted reproductive technologies (ART)”, “adenomyosis,” and “adenomyosis uteri” were used to identify all relevant articles.

### 2.2 Study selection

Two researchers, GK and TK, conducted independent manual screening of identified papers using predefined eligibility criteria, without relying on automated tools. Discrepancies between their assessments were resolved through discussion. In cases where consensus could not be reached, a third party, ID, was consulted in order to arrive at a consensus. Studies from January 2012 to 1 June 2024 in the English language were included. Study-type selection included retrospective and prospective observational and interventional studies involving a minimum of ten patients. Specific inclusion and exclusion criteria were predetermined prior to initiating the literature search. Full text articles retrieved from the above-mentioned databases were considered for inclusion in the study if they reported data on the type of adenomyosis (intrinsic or extrinsic) and at least one of the following outcomes: pregnancy rates, miscarriage rates, presence of endometriosis and outcomes of assisted reproductive technology (ART) procedures. Regarding ART procedures, all IVF protocols applied were considered eligible for inclusion. We aimed to evaluate the clinical pregnancy rate, pregnancy loss, and live birth rates in patients with adenomyosis. In the study design, a deliberate decision was made to exclude studies that involved participants with other morbidities or infertility etiologies such as pelvic inflammatory disease and male infertility factors. However, a nuanced approach was taken regarding endometriosis. Since the definitive exclusion of endometriosis without laparoscopy is challenging, the decision was made to include participants with a suspicion or clinical diagnosis of endometriosis. For this subset of patients, a subgroup analysis was applied, recognizing the need to account for the complexities and diagnostic challenges associated with endometriosis. Exclusion criteria were applied as follows: (1) Studies derived from sources other than original full publications, including reviews, abstracts, oral presentations, as well as national or local health statistics; (2) Studies not available in the English language and (3) case series with less than 10 patients were also excluded from consideration in this analysis.

### 2.3 Data extraction

Data were extracted manually from the included studies by two researchers independently. Were discrepancies occurred, they were solved by discussion and if necessary, a third researcher was consulted. Data were extracted using a standardized form to obtain the following: author, year of publication, study location, study design, number of patients, age, BMI, MRI data and/ or sonography data, adenomyosis type, data regarding the presence of endometriosis, gravidity, parity, number of miscarriages, ART outcomes; live birth rates and information regarding the stimulation protocol and number of oocytes retrieved.

**Figure 2:**
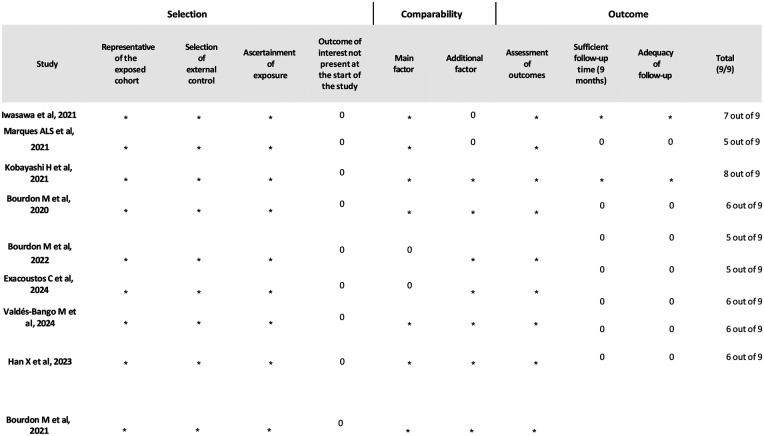
Newcastle-Ottawa scale.

The Newcastle Ottawa (http://www.ohri.ca/programs/clinical_epidemiology/oxford.asp) was used to assess the methodological quality of the included studies (Figure 2) using ten items in three domains, adapted from the Newcastle-Ottawa scale: No ethics board approval was requested, as the data were extracted from published papers.

## 3 Results

### 3.1 Studies' general characteristics

The search strategy initially identified 5,608 articles. Following title screening and removal of duplicates, the literature search yielded 9 records, which were included in the final analyses. It is important to note that two studies meeting the inclusion criteria, conducted by Mathilde Bourdon's research group, involved the same patient population. Consequently, their findings have been consolidated and presented altogether as the results of a single scientific effort (10, 13). Nine studies were included in this review, comprising three prospective cohort studies (14–16) and six retrospective studies (10, 11, 13, 17–19) All studies provided data on diagnostic methods for adenomyosis. Six studies employed MRI based on predefined criteria for diagnosis (10, 11, 13, 15, 17, 18), while three relied on ultrasound (10, 14, 16). Adenomyosis was consistently classified as intrinsic (inner myometrium) or extrinsic (outer myometrium) based on its location (Table 1).

A multicenter retrospective cohort study conducted in Japan evaluated 52 infertile patients diagnosed with adenomyosis via MRI, who underwent IVF/ICSI with fresh or frozen-thawed embryo transfer. Among these patients, 9 had extrinsic, 3 intrinsic, and 40 advanced adenomyosis (defined by the authors as full- thickness invasion with endometrial tissue) (18). There was no subclassification of adenomyosis as either focal or diffuse. There were no significant differences in age or history of uterine surgeries between groups, but patients with extrinsic adenomyosis had significantly lower BMI.

In a 2021 prospective study, Marques et al. (16) examined the correlation between sonographic signs and symptoms of adenomyosis. Among the 43 patients with adenomyosis, 22 had intrinsic, 11 extrinsic, and 10 diffuse forms. The diffuse form was diagnosed on 2D- or 3D-transvaginal ultrasound, or when the so-called question mark sign was present. The question mark sign describes a retroflexed uterine configuration in which the fundus is oriented toward the posterior pelvic compartment, while the cervix is directed anteriorly toward the urinary bladder. This definition reflects the extent of myometrial involvement and should therefore be interpreted as a severity descriptor rather than a distinct localization phenotype. Patients with the intrinsic or extrinsic form of the disease had a focal type of adenomyosis. Patients with diffuse adenomyosis were significantly older (36.7 ± 2.6 years) than those with intrinsic (30.5 ± 5.6 years) or extrinsic adenomyosis (29.4 ± 5.7 years). BMI data were not available. The next study addressing the different clinicopathological characteristics of adenomyosis subtypes is the prospective study of Kobayashi et al. (15) This study evaluated patients who underwent surgical removal of their adenomyosis because of refractory symptoms. Among the 189 patients, 78 had extrinsic, 74 intrinsic, and 37 unclassified adenomyosis. Data on whether these types were focal or diffuse are not available. No significant differences were noted in age or BMI among the groups.

Mathilde Bourdon's research group has made significant contributions to understanding the distinct subtypes of adenomyosis—intrinsic (internal) and extrinsic (external)—through two notable retrospective studies. The first study, published in 2020 (13), analyzed data from 496 patients who underwent MRI for benign indications, focusing on three cohorts: women with primary infertility, secondary infertility, and no infertility. Among these, 248 patients had MRI-confirmed adenomyosis. The study compared focal adenomyosis of the outer myometrium (FOAM, representing extrinsic adenomyosis) with internal diffuse adenomyosis, characterized by widespread foci of endometrial glands and stroma within the inner myometrium. Of the 248 patients, 109 had FOAM (extrinsic adenomyosis), 78 had intrinsic adenomyosis, and 61 had both intrinsic and extrinsic types (mixed type). Although age and BMI were not directly compared in the first publication, the group's subsequent analysis provided these insights (10). Women with extrinsic adenomyosis were younger (mean age 31.9 ± 4.6 years) than those with intrinsic adenomyosis (33.8 ± 5.2 years). Similarly, BMI was significantly lower in the extrinsic group (21.8 ± 3.13 kg/m^2^) compared to the intrinsic group (23.17 ± 4.14 kg/m^2^).

A year later, the same research group from Paris published another retrospective study focusing on patients with endometriosis who underwent MRI evaluations as part of their pre-assisted reproductive technology (ART) workup (17). This study included women aged 18 to 42 who received IVF/ICSI treatment at a tertiary care center between June 2015 and July 2018. Of the 202 participants, 145 (71.8%) had adenomyosis lesions identified on MRI. Among these, 58 women (28.7%) exhibited internal diffuse adenomyosis, while 123 (60.9%) had external adenomyosis lesions, specifically focal adenomyosis of the outer myometrium (FAOM). Additionally, 36 women (17.8%) displayed adenomyosis lesions involving both the internal and external myometrium. However, the study did not provide a direct comparison of demographic characteristics, such as age and BMI, between the adenomyosis subgroups.

A recent retrospective study by Exacoustos et al. (19) examined recurrent pregnancy loss (RPL) and its prevalence in adenomyosis patients. RPL was defined according to ESHRE guidelines, as the loss of two or more pregnancies before 24 weeks of gestation. The adenomyosis was diagnosed sonographically. Participants were divided into three groups: Group 1 (*n* = 40) included women with RPL and adenomyosis, Group 2 (*n* = 40) consisted of women with RPL but no adenomyosis, and Group 3 (*n* = 40) comprised women without RPL but with sonographic signs of adenomyosis. Among the 80 adenomyosis patients, 15 had intrinsic and 42 extrinsic forms. Among patients with intrinsic adenomyosis, 9 patients had focal adenomyosis, while 6 had diffuse adenomyosis. Among the patients with extrinsic adenomyosis,13 had a focal type, while 29 had diffuse adenomyosis.

In a single-center prospective study, Valdes et al. (14) compared clinical outcomes in 505 adenomyosis patients. Of these, 353 (69.9%) had internal and 152 (30.1%) external adenomyosis. Patients with internal adenomyosis were older (41.2 ± 6.1 years) than those with external adenomyosis (39.7 ± 6.2 years). No BMI data were reported. Among patients with intrinsic adenomyosis 13 (3.7%) patients had focal adenomyosis, while 340 (96.3%) had diffuse adenomyosis. Among the patients with extrinsic adenomyosis, 145 (95.4%) had a focal type, while 7 (4.6%) had diffuse adenomyosis.

Similarly, Han et al. (11) aimed to correlate clinical symptoms with different types of adenomyosis in 392 adenomyosis patients, identifying 184 with intrinsic and 208 with extrinsic forms. Patients with extrinsic adenomyosis were younger (36.93 ± 6.29 years) than those with intrinsic adenomyosis (42.10 ± 6.19 years), but BMI data and information on whether these types were focal or diffuse were not available.

### 3.2 Presence of endometriosis

Comparisons between extrinsic and intrinsic adenomyosis consistently demonstrate a higher prevalence of endometriosis in patients with extrinsic adenomyosis (Table 2). The first study to confirm these results is the study by Iwasawa et al. (18). There endometriosis was present in 60% (24/40) of patients in the advanced adenomyosis group and 44% (4/9) in the extrinsic group but was absent in patients with intrinsic adenomyosis. The proportion of patients who underwent ovarian endometrioma surgery was significantly higher in the extrinsic group (77.8%) compared to the advanced (32.5%) and intrinsic group of patients (33.3%). Similarly, the prevalence of ovarian endometriomas detected via MRI was 100% in the extrinsic group, 70% in the advanced group, and 0% in the intrinsic group (*p* = 0.004).

The next study to support the higher prevalence of endometriosis in extrinsic adenomyosis was the study of Kobayashi et al. (15) which found that patients with extrinsic adenomyosis had a significantly higher prevalence of deep infiltrating endometriosis and/or peritoneal endometriosis (64.1%, 50/78) compared to intrinsic adenomyosis (6.8%, 5/74). Similarly, ovarian endometriomas were more common in the extrinsic group (64.1%, 50/78) compared to the intrinsic (5.4%, 4/74) and other groups (24.3%, 9/37; *p* < 0.001). In a comparative analysis of adenomyosis subtypes, 20 patients with intrinsic adenomyosis involving the inner one-third of the myometrium (A1 group) were compared with 34 patients with extrinsic adenomyosis affecting the outer one-third of the myometrium (B1 group). The B1 group had a markedly higher prevalence of ovarian endometriomas (OMA) (51.2% vs. 5.0%, *P* < 0.001) as well as deep infiltrating endometriosis (DIE) and/or superficial peritoneal endometriosis (SUP) (51.2% vs. 0%, *P* < 0.001) when compared to the A1 group.

In the French studies by Bourdon et al. (10, 13) more severe forms of endometriosis, particularly DIE, were more prevalent in extrinsic adenomyosis (89.0%, 97/109) compared to intrinsic adenomyosis (31%, 24/77; *p* < 0.001). Additionally, women with extrinsic adenomyosis were more likely to have undergone previous endometriosis surgery than those with intrinsic adenomyosis (47.7% vs. 24%; *p* = 0.001). Multivariate analysis confirmed that DIE was independently associated with the extrinsic adenomyosis phenotype. No data regarding other types of endometriosis such as peritoneal and ovarian endometriosis as well as other causes of infertility were reported. It could also be demonstrated that endometriosis was significantly more prevalent among patients with FOAM, affecting 96.3% (105/109) of the patients compared to 62% (48/77) in those with diffuse adenomyosis (*P* < 0.001) (10). In their 2022 study, Bourdon et al. included only patients with endometriosis; however, they did not report the prevalence of the different adenomyosis subtypes within this population (17).

Valdes et al. (14) also reported a higher prevalence of deep infiltrating endometriosis in patients with extrinsic adenomyosis (86.8%, 132/152) compared to those with intrinsic adenomyosis (74.8%, 264/353). Specific involvement of the uterosacral ligaments and bladder (compartment B and FB, per #ENZIAN classification) was more frequent in extrinsic adenomyosis.

The last study in favor of the greater prevalence of endometriosis in patients with extrinsic adenomyosis is the large retrospective cohort study from China by Han et al. (11) were patients with extrinsic adenomyosis had significantly higher rates of DIE (39.9%, 83/208) compared to intrinsic adenomyosis (7.1%, 13/184). Similarly, ovarian endometriomas were more prevalent in extrinsic adenomyosis (69.7%, 145/208) compared to intrinsic adenomyosis (15.8%, 29/184). Marques et al. (16) reported that 55.8% (24/44) of adenomyosis patients had endometriosis, with 45% presenting deep infiltrating endometriosis (DIE) of the posterior compartment and 10% having ovarian endometriomas associated with posterior compartment endometriosis. However, this study did not differentiate between adenomyosis subtypes.

The same for the study of Exacoustos et al. (19), which reported endometriosis in 30% of adenomyosis patients with recurrent pregnancy loss (3 with ovarian endometriomas and 9 with DIE) but without distinguishing between adenomyosis subtypes.

### 3.3 Adenomyosis and infertility, recurrent pregnancy loss

Infertility, defined as the inability to achieve clinical pregnancy after more than one year of regular unprotected intercourse, has been investigated in several studies, highlighting differences among adenomyosis subtypes (Table 3). In the Japanese study of Iwasawa et al. all patients included in the study experienced infertility and the most common cause of infertility was endometriosis in 60% and 44% of the patients in the advanced and extrinsic groups while none of the patients with intrinsic adenomyosis experienced infertility due to endometriosis. Moreover, no significant differences were observed among the three groups regarding other causes of infertility (18). Uterine fibroids or congenital uterine malformations were not reported as potential causes of infertility and were likely absent.

In the prospective study by Marques et al. (16), infertility was observed in 72.7% (16/22) of patients with intrinsic adenomyosis, 81.8% (9/11) with extrinsic adenomyosis, and in all patients with diffuse adenomyosis, with statistically significant results, when diffuse adenomyosis was compared with intrinsic and extrinsic. Among patients with adenomyosis, 13 (30.2%) had concomitant uterine fibroids; however, the distribution of fibroids across the two adenomyosis subgroups was not available.

A significant contribution in understanding the distinct types of intrinsic (internal) and extrinsic (external) adenomyosis has been made by Mathilde Bourdon's research group. In the first study it could be shown that among primary infertility cases, the prevalence of focal adenomyosis of the outer myometrium (FOAM) was significantly higher; 33.3% (28/84) (adjusted odds ratio 1.9; 95% confidence interval 1.1– 3.3)., while diffuse adenomyosis was present in 8.3% (7/84). For secondary infertility, the same prevalence; 17.6% (9/51) was seen in patients with FOAM and diffuse adenomyosis. No significant differences in the presence of endometriosis or leiomyomas were observed between primary and secondary infertility cohorts. Uterine malformations were not reported as potential causes of infertility and were likely absent. Logistic regression adjusting for age, adenomyosis type, endometriosis, and leiomyomas identified FOAM as an independent factor associated with primary infertility, with an adjusted odds ratio of 1.9 (95% CI: 1.1–3.3) (13). In the subsequent French study, infertility was again found to be more common in the extrinsic group, affecting 34% (38/109) compared to 20.5% (16/77) in the intrinsic group. Primary infertility was particularly prominent in the extrinsic group, seen in 25% of cases vs. 9.0% in the intrinsic group (*P* = 0.021) (10). Bourdon et al. further demonstrated that 74.3% (81/109) of women with extrinsic adenomyosis had no prior pregnancies, compared to 33% (26/77) in the intrinsic group (*P* < 0.001) (10). These findings underscore the significant impact of FOAM and extrinsic adenomyosis on fertility outcomes. However, the 2022 study does not provide information on the presence of primary or secondary infertility based on the location of adenomyosis within the myometrium (17).

In the large retrospective Chinese cohort, infertility was significantly more prevalent in patients with extrinsic adenomyosis, affecting 41.3% (86/208) compared to 20.7% (38/184) of intrinsic adenomyosis cases (*P* < 0.05). Furthermore, primary infertility was more common in the extrinsic group (58.1%, 50/86), whereas secondary infertility predominated in the intrinsic group (76.3%, 29/38) (11). The coincidence of uterine fibroids was similar between intrinsic and extrinsic adenomyosis subgroups. Patients with fibroids >5 cm and uterine malformations were excluded from the study.

Valdes et al. found no significant difference in infertility rates between intrinsic and extrinsic adenomyosis groups, with 45.3% (160/353) of intrinsic and 48.7% (74/152) of extrinsic cases meeting the definition of infertility (14). Similarly, Kobayashi et al. found no significant differences in infertility rates between intrinsic and extrinsic adenomyosis groups or between A1 (inner 1/3 of the uterine myometrium) and B1 (outer 1/3 of the uterine myometrium) subgroups (15) In both studies there was no statistical difference in the prevalence of myomas between the intrinsic and extrinsic adenomyosis subgroups. Uterine malformations are not documented as a possible cause of infertility.

Regarding recurrent pregnancy loss (RPL), intrinsic adenomyosis was more frequently observed in patients with RPL, while extrinsic adenomyosis was more common in those without RPL. Specifically, focal adenomyosis of the inner myometrium was more prevalent among women with RPL, whereas extrinsic focal adenomyosis was more typical in those without RPL. There were no significant differences in the prevalence of diffuse adenomyosis or in the severity of adenomyosis (mild, moderate, or severe) between groups (19). Among patients with adenomyosis, 50% had coexisting fibroids, while 20% presented with uterine malformations. Specifically, 10% had a dysmorphic uterus (U1) and 10% a septate uterus (U2), according to the ESHRE/ESGE classification. However, the prevalence of fibroids and uterine malformations across the different adenomyosis subtypes has not been reported.

These studies collectively highlight the complex interplay between adenomyosis subtypes and infertility, with extrinsic adenomyosis often associated with worse fertility outcomes and a higher prevalence of primary infertility. Because some cases of adenomyosis appeared to originate in the context of endometriosis, a potential confounding influence of endometriosis on fertility and pregnancy outcomes cannot be excluded. Moreover, in patients who did not underwent surgery, the presence of concomitant endometriosis cannot be definitively ruled out.

### 3.4 Adenomyosis and ART outcomes

Three of the included studies reported data on assisted reproduction technology (ART) outcomes on the different adenomyosis subtypes (Table 4). In the study by Iwasawa et al. (18), no significant differences were observed between intrinsic and extrinsic adenomyosis groups regarding IVF or ICSI utilization, the number of embryo transfer cycles, or the median number of embryos transferred per cycle (median *N* = 1, range 1–2). The proportions of fresh vs. frozen-thawed embryo transfers were also similar across groups. Good-quality embryos were transferred in 100% of cases in the intrinsic group, 78% in the advanced group, and 63% in the extrinsic group. Pre-embryo transfer GnRH administration varied among groups, with 20% of patients in the advanced group, 3.7% in the extrinsic group, and none in the intrinsic group receiving GnRH before embryo transfer. Pregnancy loss per clinical pregnancy and live birth rates were reported as followed: 64% (16/25) and 9% (9/100) in the advanced group, 33.3% (3/9) and 22.2% (6/27) in the extrinsic group, and 50% (1/2) and 11.1% (1/9) in the intrinsic group. Logistic regression analysis, adjusted for age, prior miscarriage, and BMI, revealed that the extrinsic group had significantly fewer pregnancy losses [odds ratio (OR) 0.06; 95% CI 0.00–0.54; *P* = 0.026] and higher live birth rates (OR 6.05; 95% CI 1.41–29.65; *P* = 0.018) compared to the advanced group. Due to limited sample sizes, direct comparisons between the intrinsic and extrinsic groups were not possible.

In the Chinese study, among infertile patients undergoing ART, 12/38 (31.6%) from the intrinsic group and 27/86 (31.4%) from the extrinsic group proceeded with ART. The success rates were 25% (3/12) in the intrinsic group and 51.9% (14/27) in the extrinsic group. While the success rate was higher in the extrinsic subtype, although the difference was not statistically significant (11). Unfortunately, data regarding the IVF protocol, the number of fresh vs. frozen thawed embryos, and the quality of the embryos were not available.

Bourdon et al. (17) could demonstrate that live birth rates following ART were lower in women with adenomyosis and endometriosis (52.4%, 76/145) compared to those with endometriosis alone (70.2%, 40/57; *P* = 0.02). A total of 346 IVF/ICSI cycles were analyzed, with an average of 1.7 ± 0.8 cycles per woman. The live birth rate (LBR) after the first cycle was 37.6% (76/202). Among women with internal adenomyosis, 35 out of 64 achieved a live birth, while 29 did not. In the external adenomyosis group, 56 out of 123 experienced a live birth, while 67 did not. Multivariate analysis identified adenomyosis lesions (intrinsic and/or extrinsic) (OR 0.48; 95% CI 0.29–0.99) and T2 high-signal intensity myometrial spots (OR 0.43; 95% CI 0.22–0.86) as independent factors associated with reduced LBR (17). However, no direct comparison has been made between the different adenomyosis subtypes. Data on IVF protocols, fresh and frozen–thawed embryo transfers, and embryo quality were not available for subgroup analysis.

The analysis of ART-related outcomes in this study remains limited in scope, as it does not comprehensively address potential differences between fresh and frozen–thawed embryo transfer cycles, the different ovulation induction protocols for different adenomyosis subtypes, or the interaction between embryo quality and adenomyosis location. Due to the small number of available studies and heterogeneity of the studies providing such data, a complementary analysis could not be conducted underscoring the necessity for further research specifically addressing ART techniques to enable more reliable conclusions.

### 3.5 Adenomyosis and perinatal, pregnancy outcomes

The study of Iwasawa et al. (18) is the only one to report analytically data on perinatal and pregnancy outcomes (Table 5). In total sixteen patients had live births. Caesarean sections were performed in 62.5% of cases (10 patients, 7 with advanced and 3 with extrinsic adenomyosis), while preterm labor occurred in 18.6% (3 patients, 1 with advanced and 2 with extrinsic adenomyosis) and threatened premature labor in 50% (8 patients, 5 with advanced and 3 with extrinsic adenomyosis). One patient with an advanced type of lesion experienced placenta previa, fetal growth restriction, and preeclampsia. There were no significant differences in perinatal outcomes based on the location of adenomyosis, although safe conclusions cannot be made, because of the small number of patients.

On the study of Kobayashi et al. (15) caesarean section rates where not statistically different between the intrinsic and extrinsic types of adenomyosis.

### 3.6 Myometrial location of adenomyosis and associated outcomes

A schematic overview of adenomyosis subtypes according to myometrial location (intrinsic and extrinsic) and their corresponding outcomes is presented below (Table 6).

### 3.7 Predictors of adenomyosis subtypes

In the study by Kobayashi et al. (15), univariate analysis identified three factors as predictors of early- stage extrinsic adenomyosis: the presence of deep infiltrating endometriosis (DIE) and/or superficial peritoneal endometriosis (SUP), the presence of ovarian endometriomas (OMA), and the absence of a prior history of induced abortion (*P* < 0.001, *P* < 0.001, and *P* = 0.017, respectively). These associations were confirmed in multivariate analysis. The A1 group demonstrated a significantly higher rate of induced abortions compared to the B1 group (45.0% vs. 14.0%, *P* = 0.017). For intrinsic adenomyosis, a prior history of induced abortion and curettage emerged as significant risk factors in univariate analysis.

The French studies by Bourdon et al. (10, 13) found that DIE was independently associated with the external adenomyosis phenotype in multivariate analysis. Conversely, intrinsic adenomyosis was significantly associated with gravidity of two or more and a BMI of 30 or higher. Additionally, prior uterine surgeries, such as myomectomy, hysteroscopy, or cesarean section, were more frequently reported in the intrinsic adenomyosis group (22%) compared to the external group (6.4%), with statistically significant results.

Valdes et al. (14) reported lower nulliparity rates in intrinsic adenomyosis patients (49.0%, 173/353) compared to those with extrinsic adenomyosis (61.2%, 93/152). However, patients with extrinsic adenomyosis were younger and significantly more likely to be nulliparous compared to the intrinsic adenomyosis group. Multivariate analysis identified DIE and nulliparity as independent predictors of extrinsic adenomyosis, with adjusted odds ratios (aOR) of 2.31 (95% CI: 1.35–3.95, *P* = 0.002) and 1.67 (95% CI: 1.10–2.53, *P* = 0.016), respectively. In contrast, the presence of leiomyomas was strongly associated with intrinsic adenomyosis (aOR: 2.63; 95% CI: 1.49–4.54, *P* = 0.001). Myomas were observed in (25.3%, 89/353) patients with intrinsic adenomyosis compared 13.2% of patients with extrinsic adenomyosis. These findings align with the French studies by Bourdon et al., which also identified a higher prevalence of leiomyomas in the internal adenomyosis group compared to the external adenomyosis group [14 (18%) vs. 3 (2.8%); P.0.001] (10). These observations underline the distinct clinical and pathological profiles of intrinsic and extrinsic adenomyosis, emphasizing the role of associated comorbidities in the formation of adenomyosis.

## 4 Discussion

The diversity of adenomyotic presentations has led to a growing interest in understanding its distinct types, particularly intrinsic and extrinsic adenomyosis, and their unique clinical and pathological characteristics. The impact of adenomyosis localization within the myometrium on fertility and pregnancy outcomes is a subject of increasing clinical and research interest. The classification of adenomyosis into intrinsic and extrinsic subtypes has provided insights into the pathophysiological mechanisms affecting reproductive outcomes.

The narrative systematic review analyzed nine studies, including both prospective and retrospective studies, that assessed the diagnostic accuracy of MRI and ultrasound in differentiating intrinsic from extrinsic adenomyosis. In the existing literature, terms describing adenomyosis localization are frequently used interchangeably with descriptions of disease extent or severity, which may generate conceptual ambiguity.

Types such as “advanced” or “diffuse” adenomyosis, reported in the study of Iwasawa (18) and Marques (16), should not be interpreted as distinct localization phenotypes. Rather, these terms primarily reflect the spatial extent or severity of myometrial involvement. Accordingly, in the present review, adenomyosis is conceptualized using a two-dimensional framework: (i) location phenotype, defined as intrinsic (inner myometrium), extrinsic (outer myometrium), or true mixed disease when both patterns coexist; and (ii) extent/severity, described as focal vs. diffuse. Where full-thickness or “advanced” disease is reported, there is interpreted as a study-specific descriptor rather than a universal localization category. A further limitation inherent to the current body of evidence is the unclear interpretation of the “mixed” adenomyosis phenotype. Across published studies, this category may encompass fundamentally different pathological scenarios, including (i) the true coexistence of intrinsic and extrinsic lesions, and (ii) late-stage or extensive disease characterized by diffuse or full-thickness myometrial involvement that produces a “pseudo-mixed” imaging appearance. Because most studies do not apply standardized criteria to distinguish between these entities, the mixed phenotype remains inconsistently defined, increasing the risk of misclassification and limiting the interpretability of phenotype–outcome associations.

As far as we are concerned the mixed adenomyosis phenotype should be defined as the true coexistence of intrinsic and extrinsic disease, with discrete involvement of both the inner myometrium/junctional zone and the outer myometrium/serosal side, as also described from Kishi in 2012 (20). From a practical imaging perspective, magnetic resonance imaging and ultrasound can aid this distinction by assessing predominant junctional zone or inner myometrial involvement for intrinsic disease, vs. serosal-side or outer myometrial infiltration, often in continuity with deep infiltrating endometriosis for extrinsic disease. Recognition of these patterns is essential to avoid misclassification of advanced, extensive disease as a true mixed phenotype and to improve the consistency of phenotype-based interpretation across studies.

The significant heterogeneity among the studies prevented a formal meta-analysis, but important trends emerged regarding the association of each adenomyosis subtype with infertility and pregnancy loss. Extrinsic adenomyosis was consistently linked to a higher prevalence of endometriosis, particularly DIE and ovarian endometriomas, with studies reporting an association rate of up to 89%. This subtype seemed also to be associated with a higher prevalence of primary infertility, with rates reaching 41.3% compared to 20.7% in intrinsic adenomyosis. Conversely, intrinsic adenomyosis showed A stronger link with recurrent pregnancy loss (RPL) and secondary infertility. In patients presenting with both adenomyosis and endometriosis, the extent to which these conditions interact to influence pregnancy outcomes—potentially leading to poorer results than those observed with a single pathology—has yet to be determined.

Regarding assisted reproductive technology (ART) outcomes, conclusions cannot be made due to the limited number of the available data and heterogeneity of the studies. The absence of stratified analyses including the number of ART cycles, oocyte retrieval numbers, and fertilization rates across studies, limits the ability to determine whether the effect of adenomyosis subtypes on ART outcomes is mediated by factors such as ovarian response or oocyte quantity and quality. However, subtypes demonstrated lower live birth rates compared to control groups without adenomyosis or endometriosis. This highlights the need for further investigation into individualized treatment strategies to improve ART outcomes in affected patients.

Identifying risk factors and predictors for different adenomyosis subtypes is crucial for optimizing fertility treatments and pregnancy management. Extrinsic adenomyosis was more prevalent in younger women, nulliparous individuals, and those with DIE or ovarian endometriomas. In contrast, intrinsic adenomyosis was associated with prior uterine surgeries (e.g., myomectomy, cesarean section), higher BMI, and multiparity. Furthermore, the frequent coexistence of leiomyomas with intrinsic adenomyosis underscores the complex interplay between uterine structural abnormalities and reproductive outcomes. A crucial aspect of reproductive success is the integrity of the junctional zone, which plays a pivotal role in decidualization, implantation, and placental development. Structural disruptions in the JZ due to intrinsic adenomyosis may lead to impaired blastocyst invasion and abnormal vascular remodeling of spiral arteries, ultimately increasing the risk of miscarriage and obstetric complications such as preeclampsia and preterm birth. The findings of Exacoustos et al. (19) highlight the potential of intrinsic adenomyosis to compromise uterine peristalsis and inflammatory homeostasis, further contributing to adverse pregnancy outcomes.

This narrative systematic review included studies of medium quality, which presents certain limitations. Due to the limited sample size, further subclassification of adenomyosis into focal and diffuse forms could not be performed. To our knowledge, this aspect remains insufficiently explored in the current literature and warrants further investigation, particularly as different adenomyosis phenotypes may have distinct implications for fertility and pregnancy outcomes. At present, the available literature does not provide standardized or validated clinical or imaging criteria that define severity-related descriptors such as “diffuse” or “advanced” adenomyosis as a distinct classification axis independent of lesion localization. Existing studies apply heterogeneous and often study- specific criteria, which limits comparability and precludes firm conclusions regarding severity-based phenotyping. In parallel, there is no widely accepted consensus on how to reliably distinguish a true mixed localization phenotype, reflecting the coexistence of intrinsic and extrinsic disease from pseudo-mixed appearances arising in late-stage, extensive myometrial involvement. This conceptual and methodological variability represents an inherent limitation of the current evidence and underscores the need for harmonized definitions in future research. Additionally, the retrospective nature and small number of patients of some studies may have led to selection bias, and variations in patient populations across studies further complicated direct comparisons. Despite these limitations we could saw the different heterogeneous nature of adenomyosis and to clarify how the distinct phenotypes of intrinsic and extrinsic adenomyosis may uniquely impact reproductive health and pregnancy outcomes.

## 5 Conclusion

Intrinsic adenomyosis, which primarily involves the inner myometrium and junctional zone (JZ), appears to have distinct implications for embryo implantation and pregnancy maintenance compared to extrinsic adenomyosis, which affects the outer myometrium and is more commonly associated with deep infiltrating endometriosis (DIE). Future research should aim to standardize diagnostic protocols for distinguishing intrinsic and extrinsic adenomyosis, the mixed type and their subtypes like focal and diffuse incorporating advanced imaging techniques. Large- scale, high-quality prospective cohort studies and randomized controlled trials are needed to better understand the impact of adenomyosis subtypes on fertility and pregnancy outcomes.

## Data Availability

The raw data supporting the conclusions of this article will be made available by the authors, without undue reservation.
